# Extracellular vesicles released by LPS-stimulated spinal organotypic slices spread neuroinflammation into naïve slices through connexin43 hemichannel opening and astrocyte aberrant calcium dynamics

**DOI:** 10.3389/fncel.2024.1433309

**Published:** 2024-07-10

**Authors:** Christian Memo, Pietro Parisse, Roberta Amoriello, Maria Pachetti, Anabela Palandri, Loredana Casalis, Clara Ballerini, Laura Ballerini

**Affiliations:** ^1^Neuroscience Area, International School for Advanced Studies (SISSA/ISAS), Trieste, Italy; ^2^Nanoinnovation Lab, ELETTRA Synchrotron Light Source, Basovizza, Italy; ^3^CNR-IOM, Basovizza, Italy; ^4^Dipartimento di Medicina Sperimentale e Clinica, University of Florence, Firenze, Italy

**Keywords:** atomic force microscopy, calcium imaging, GAP27, cytokine and chemokine, neuroglia and inflammation

## Abstract

**Introduction:**

Neuroinflammation is a hallmark of multiple neurodegenerative diseases, shared by all pathological processes which primarily impact on neurons, including Central Nervous System (CNS) injuries. In reactive CNS, activated glia releases extracellular vesicles (EVs), nanosized membranous particles known to play a key role in intercellular communication. EVs mediate neuroinflammatory responses and might exacerbate tissue deterioration, ultimately influencing neurodegenerative disease progression.

**Methods:**

We treated spinal cord organotypic slices with LPS, a ligand extensively used to induce sEVs release, to mimic mild inflammatory conditions. We combine atomic force microscopy (AFM), nanoparticle tracking (NTA) and western blot (WB) analysis to validate the isolation and characterisation of sEVs. We further use immunofluorescence and confocal microscopy with live calcium imaging by GCaMP6f reporter to compare glial reactivity to treatments with sEVs when isolated from resting and LPS treated organ slices.

**Results:**

In our study, we focus on CNS released small EVs (sEVs) and their impact on the biology of inflammatory environment. We address sEVs local signalling within the CNS tissue, in particular their involvement in inflammation spreading mechanism(s). sEVs are harvested from mouse organotypic spinal cord cultures, an in vitro model which features 3D complexity and retains spinal cord resident cells. By confocal microscopy and live calcium imaging we monitor glial responses in naïve spinal slices when exposed to sEVs isolated from resting and LPS treated organ slices.

**Discussion:**

We show that sEVs, only when released during LPS neuroinflammation, recruit naïve astrocytes in the neuroinflammation cycle and we propose that such recruitment be mediated by EVs hemichannel (HC) permeability.

## 1 Introduction

Extracellular vesicles (EVs) are heterogeneous membranous particles released virtually by any cell phenotype to mediate intercellular signaling in multiple physiological and pathological processes (Dolcetti et al., 2020). EVs modulate specific functions of the recipient cells through membrane expression and/or delivery of signaling molecules, such as microRNA (miRNA), messenger RNA (mRNA), lipids, proteins, or other bioactive molecules (Pan and Johnstone, 1983; Rashed et al., 2017; Soria et al., 2017; Doyle and Wang, 2019).

In the central nervous system (CNS), EVs can be secreted by both neuronal and non-neuronal cells to promote communication among heterogeneous cell populations. Glial cells, such as astrocytes, microglia, and oligodendrocytes, release EVs to contribute to CNS homeostasis (Venturini et al., 2019) in a healthy context, or when released by reactive glia in response to disease processes, EVs might favor degenerative or inflammatory escalation (Marostica et al., 2021; Zhao et al., 2021). For instance, in neurodegenerative diseases, astrocyte-derived EVs were reported to contribute to proinflammatory signaling, ultimately exacerbating tissue reactivity and neuronal damage (Goetzl et al., 2016; Oyarce et al., 2022).

Overall, EVs seem to have a key role in disease progression in the CNS (Delpech et al., 2019) by propagating cellular reactivity and neuroinflammatory responses. In the context of neuroinflammation, further investigations are needed to underscore both the efficiency of glia-released EVs in promoting neuroinflammation progression and the molecular mechanisms involved in the recruiting of naïve astrocytes to spread tissue reactivity. The results may lead to novel therapeutic targets, with the aim to mitigate the neuroinflammatory vicious cycle.

In this study, we focused on the smaller sub-class of EVs secreted by the fusion of the multivesicular body with the plasma membrane (Delpech et al., 2019) identified as small EVs (sEVs), which is less than 200 nm in diameter (Möller and Lobb, 2020). To isolate and explore sEV release and activity during CNS inflammation, we adopted a slice *in vitro* model, the organotypic spinal cord cultures, which retain the spinal sensory-motor cytoarchitecture with segment ventral–dorsal organization, allowing direct experimental access to spinal resident cells (Furlan et al., 2007; Medelin et al., 2008). This model has allowed exploring neuronal and non-neuronal responses to inflammation when experimentally induced by different danger signals (Giacco et al., 2019; Panattoni et al., 2021), with a recent focus on the active role of astrocytes in CNS reactivity progression (Panattoni et al., 2021; Di Mauro et al., 2023).

We mimicked mild inflammatory conditions by treating spinal slices with LPS, a ligand extensively used to induce the secretion of sEVs (Delpech et al., 2019). LPS has been reported to mediate spinal reactivity with an increase in cytokine and chemokine production combined with microglia and astrocyte activation (Panattoni et al., 2021). sEVs were harvested from resting and LPS-reactive cultures and were characterized by AFM, NTA, and WB analysis. We combined immunofluorescence and confocal microscopy with live calcium imaging by GCaMP6f reporter to compare glial reactivity with treatments with sEVs when isolated from resting and LPS-treated organ slices. sEVs, released during LPS neuroinflammation, propagate reactive responses in naïve spinal slices, which are characterized by reactive astrocytes displaying aberrant calcium dynamics. We further show that the recruitment of naïve astrocytes in sEVs in response to neuroinflammation is mediated by increased hemichannel (HC) permeability.

## 2 Materials and methods

### 2.1 Organotypic spinal cord cultures, benzoyl-ATP and LPS treatments, and sEV isolation

All experiments were performed in accordance with the EU guidelines (2010/63/UE) and Italian law (Decree 26/14) and were approved by the local authority veterinary service and our institution (SISSA) animal wellbeing committee (OBPA). All efforts were made to minimize animal suffering and reduce the number of animals used. The use of animals was approved by the Italian Ministry of Health (no. 22DABNQYA and 22DABN1WO) in agreement with the EU Recommendation 2007/526/CE. Organotypic spinal cord and dorsal root ganglia (DRG) slices (275 μm) were isolated from C57BL/6 J mouse embryos (embryonic days E12–13) and cultured in plastic tubes containing 1 mL of medium as previously described (Avossa et al., 2003; Medelin et al., 2008; Giacco et al., 2019; Panattoni et al., 2021). The tubes were kept in a roller drum rotating 120 times per hour in an incubator at 37°C in the presence of a humidified atmosphere with 5% CO_2_. All the experiments were performed on spinal slice cultures at 2–3 weeks *in vitro* (WIV).

To isolate sEVs released from spinal cords in organ cultures, we collected the supernatant from *n* = 12 spinal cord-cultured slices, which was previously maintained (24 h) in a culture medium enriched with sEV-depleted FBS (named depleted medium). According to MISEV2018 (Théry et al., 2018), sEVs were removed from FBS by ultracentrifugation for 18 h at 100,000 × g at 4°C.

Before sEV isolation, cultures were treated with benzoyl-ATP (bzATP 100 μM, 1 h; Sigma) for a positive sEV secretion control (Delpech et al., 2019; Musto et al., 2021), with depleted medium for basal sEV secretion control and LPS (LPS 1 μg/mL, 24 h; Sigma, O55:B5) for sEV secretion in inflammation condition (Giacco et al., 2019). We extracted sEVs in each group by differential centrifugation of the harvested supernatants at 4°C, first 15 min at 300 × g followed by 15 min at 3000 × g and then 70 min at 20000 × g in order to remove cell debris, apoptotic bodies, microvesicles, and other larger particles. The resulting supernatant was finally filtered with 0.22-μm syringe filters (Merck Millipore) and ultracentrifuged in a Beckman Coulter Optima™ XE90 Ultracentrifuge for 70 min at 120,000 × g using a SW 41 Ti rotor to pellet sEVs (adapted from the study by Théry et al., 2006; Musto et al., 2021).

The pellets were resuspended in 500 μL of PBS for sEV characterization (see below) or in 500 μL of depleted medium for slice treatments (see below).

### 2.2 Atomic force microscopy analysis

Atomic force microscopy (AFM) characterization was performed as previously described (Musto et al., 2021). In brief, 15 μL of freshly isolated sEVs resuspended in PBS solution was placed and left to adsorb (30 min) onto a freshly peeled mica substrate. AFM analysis was performed in liquid at room temperature (RT) using the AC mode of a commercial instrument (MFP-3d, Asylum Research/Oxford Instruments, United Kingdom). Silicon nitride cantilevers with sharp (<10 nm tip radius) silicon tips (BL-AC40-TS, Olympus, JP), a typical force constant of 0.09 nN/nm, and a resonance frequency of 30 kHz in liquid were used. Topographic height and phase images were recorded at 512 × 512 pixels at a scan rate of 0.5 Hz. Image processing was performed using *Gwyddion freeware* AFM analysis software (version 2.40). Diameter and height of each vesicle were evaluated from cross-line profiles, and the results were statistically analyzed using Prism (*Graphpad* software).

### 2.3 Nanoparticle tracking analysis

Measurement and analysis of sEV size distribution by NTA were performed by a NanoSight LM10 system (Malvern) using 500 μL of sEVs in PBS solution for each of the three groups (Control sEVs, bzATP sEVs, and LPS sEVs) and diluted to 1:100 in MilliQ H_2_O. Individual videos of 60 s (recorded at 25 FPS; three videos for each group, control, bzATP, and LPS) were acquired at RT using the maximum camera gain and a detector threshold equal to 5 and analyzed by the NanoSight particle tracking software to calculate the size and concentration of vesicles.

### 2.4 Western blot analysis

Freshly isolated sEVs were resuspended in lysis buffer (50 mM Tris–HCl, pH 8.0, 150 mM NaCl, 1% NP40, 0.1% SDS), added with protease inhibitor cocktail (Sigma), sonicated for 30 s, and then boiled at 95°C for 5 min. Samples were run on 8–10% polyacrylamide gel and blotted onto PVDF membranes (Millipore, Italy). Membranes were then blocked in TBS plus 5% non-fat dry milk (Sigma) for 1 h and incubated overnight at 4°C (in shacking condition) with anti-flotillin1 (FLO-1, dilution 1:1000, 610,821, BD Biosciences), anti-tumor susceptibility 101 (TSG101, dilution 1:1000, PA5-31260, Invitrogen), anti-golgi matrix protein 130 (GM130, dilution 1:1000, PA5-95727, Invitrogen), and anti-connexin-43 (Cx43, dilution 1:500, ab11370, Abcam) primary antibodies.

Membranes were then washed with TBS-Tween-20 (0.1%) and incubated with peroxidase-conjugated anti-mouse and anti-rabbit secondary antibodies (dilution 1:1000) for 2 h at RT. Protein bands were visualized using the chemiluminescence detection system (UVI-1D software).

### 2.5 Analysis of cytokines and chemokines by Luminex assay

In the supernatant of organotypic slices, a panel of 13 cytokines and chemokines was analyzed (IL1α, IL1β, IL4, IL6, IL10, IL12p40, IL12p70, IL17, IFNγ, C-X-C motif ligand 10 [CXCL10], C-C motif ligand 2 [CCL2], C-X-C motif ligand 2 [CXCL2], and TNFα) by Milliplex assay (Merck Millipore, United States, #MCYTOMAG-70 K) and Bio-Plex device (Bio-Rad, USA), as previously described (Di Mauro et al., 2023). The amount of cytokines and chemokines was expressed as pg./ml. The detection threshold was 1.0 pg./mL for all the analyzed factors. We used three replicates for three organotypic cultures, corresponding to nine measurements for each experimental group.

### 2.6 Treatment of naïve organotypic slices

For experiments involving chronic sEV treatments, organotypic cultures (2–3 WIV) were incubated (24 h) in a depleted medium alone as control or in a depleted medium with: (i) LPS as reference inflammation condition, (ii) sEVs isolated from controls, and (iii) sEVs isolated from LPS-treated slices. In conditions (ii) and (iii), sEVs were resuspended in halved medium volume (i.e., sEVs extracted from 10 mL of medium were resuspended in 5 mL of treatment medium).

In an additional set of experiments (reported in the Supplementary material), after sEVs were re-isolated and removed, naïve slices were incubated (24 h) with the supernatant derived from the LPS treatment medium (upon removal of sEVs), and the fresh medium was used to resuspend sEVs. All treatments (LPS or sEVs) were washed out prior to immunostaining analysis or live cell imaging recordings.

### 2.7 Immunofluorescence imaging, Lucifer yellow live uptake, and analysis

For immunostaining, organotypic cultures were fixed with 4% paraformaldehyde (PFA, prepared from fresh paraformaldehyde; Sigma) in PBS (1 ×) for 1 h (RT) and then washed with PBS. Free aldehyde groups were quenched in sodium borohydride (NaBH4, Sigma) 1% in PBS for 5 min. Slices were permeabilized and blocked in PBS (1 ×), 5% FBS (Sigma), 1% BSA (Sigma), and 0.3% Triton X100 (Sigma) at RT for 1 h and incubated overnight at 4°C with anti-GFAP (mouse monoclonal, 1:200, Sigma) and anti-IBA1 (rabbit polyclonal, 1:500, Wako) primary antibodies. Subsequently, the slices were PBS-washed, incubated with secondary antibodies (Alexa 488 goat anti-mouse and Alexa 594 goat anti-rabbit, 1:500 dilution, Invitrogen), and diluted in blocking solution for 2 h (RT) in the dark. DAPI (1:500 dilution, Thermo Fisher Scientific) was used to stain the nuclei. Samples were mounted on glass coverslips using Fluoromount-G aqueous mounting medium (Thermo Fisher Scientific). Images were acquired using a Nikon A1 Confocal microscope with Ar/Kr, He/Ne, and UV laser with 20 × or 40 × objective (0.95 N.A.). Confocal sections were acquired up to a z-stack thickness of 5–15 μm (depending on the required analysis) in sequential mode.

For each experiment, we performed ≥ 3 independent culture series; from each culture series, we analyzed ≥ 3 slices for each condition, and from each slice, ≥ 3 fields were randomly acquired from the ventral region of the spinal slices. Offline analysis of the image z-stack was performed using *Volocity 3D* image analysis software. We performed offline analysis of the z-stack for calculating the Transformation Index (TI), which categorized microglia ramification status, and the GFAP signal volume. In brief, to quantify microglia ramification degree as a correlation with changes in the reactive state, we calculate TI as follows: [perimeter of the cell (μm)]^2^/4π[area of the cell (μm^2^)] (Fujita et al., 1996; Giacco et al., 2019). A cell with long processes and a small soma exhibits a large index which is dependent on cell shape but independent of cell size.

In every acquired field, astrocyte reactivity was quantified by measuring the volume occupied by the GFAP signal in the z-stack, which was calculated by the calibrated software and was expressed in μm^3^.

To measure the opening of connexin HCs in astrocytes, we quantify the Lucifer Yellow (LY; Sigma) uptake in GFAP-positive cells. Cultures were incubated at 37°C with 1 mM LY in standard physiological solution for 10 min and then washed three times with the same solution without LY (Chepied et al., 2020). Cultures were fixed with PFA, and subsequently, astrocytes were labeled using an anti-GFAP antibody as described above. Images were acquired at the confocal microscope using 20 × and 40 × objectives. Confocal sections were acquired from 1 μm to z-stack thickness of 5 μm. The analysis of LY-GFAP double-positive astrocytes was performed using the “Intersect objects” tool within *Volocity* software (Giacco et al., 2019), to quantify only the objects where LY and GFAP signals were intersected. A size threshold limit was set to exclude all the objects with a dimension of <5 μm^3^ in order to avoid background signal interference (Panattoni et al., 2021). The results were expressed as the mean number of LY/GFAP double-positive cells for each treatment condition. LY-positive cell density (plot in Supplementary Figure S2B) was quantified as LY-positive cells per field (40 × fluorescent micrograph) by using the “Find Maxima” plug-in in Fiji ImageJ software. Pearson correlation coefficient (r) was calculated using “Coloc 2” plug-in of Fiji ImageJ software between GFAP and sEV_LPS_ carrying Cx43 tagged with GAP27 + Alexa Fluor 488 NHS Ester signals.

### 2.8 Calcium imaging with GCaMP6f and analysis

Organotypic slices at DIV 9 were infected with AAV5.gfaABC1D-cyto-GCaMP6f (#52925-AAV5, Addgene), which leads to a selective expression of the genetically encoded calcium indicator (GECI) GCaMP6f in astrocytes. At 3 WIV, before the recording procedures, cultures were treated for 24 h with depleted medium as a control condition, with LPS and sEVs extracted after LPS treatment. On the day of the recording, the slices were maintained in an incubator for habituation for 15 min, immersed in extracellular saline solution, and also used as a recording solution, which was composed of (mM) 150 NaCl, 4 KCl, 1 MgCl_2_, 2 CaCl_2_, 10 HEPES, and 10 Glucose (pH adjusted to 7.35 with 2 M NaOH). Slices were then placed in a custom 3D-printed perfusion chamber and mounted on an inverted Nikon microscope (Nikon Eclipse Ti2 microscope endowed with a Nikon IntensiLight Hg lamp and an Andor Zyla sCMOS camera), where they were continuously superfused (5 mL/min, ≥25 min) at RT with the recording saline solution. GCaMP6f fluorescence was monitored with a 20 × S Plan Fluor ELWD (NA 0.45) objective from the ventral horn area. Images were taken with an exposure time of 150 ms and 4 × 4 binning. The acquired time series of images were analyzed in Fiji choosing ROIs of ~ Ø 20–30 μm, and the fluorescence traces were processed in ClampFit 10.7 (Panattoni et al., 2021).

Ca^2+^ transients are expressed as ΔF, where ΔF is the fluorescence rise over baseline, which is calculated as follows: *F − F_0_*. *F_0_* (baseline fluorescence) is calculated as the median of the frame fluorescence values.

Ca^2+^ transients were analyzed using the inter-event interval (IEI) parameter, which measured the period between successive peaks of calcium concentration within a cell, particularly informative when calcium activity is irregular (i.e., when bursts of activity are present) or occurrs at a low pace.

### 2.9 GAP27 treatment of sEVs

Freshly isolated sEVs from LPS-treated slices were incubated with the Cx43 inhibitor peptide GAP27 (GeneCust; Panattoni et al., 2021) at a concentration of 500 μM for 1 h at 4°C under constant agitation using a rotating mixer. To remove the excess of GAP27 peptide after the incubation period, sEVs were resuspended in 10 mL of depleted medium, pelleted again for 70 min at 120,000 × g, and resuspended in 500 μL of depleted medium ready to be used for treatments.

### 2.10 sEVs Cx43 tagging

GAP27 has been conjugated with Alexa Fluor 488 NHS Ester (Invitrogen; Cat. number: A20000) by incubation, according to the manufacturer’s instructions. After incubation, the excess of unbounded Alexa Fluor 488 NHS Ester has been removed by dialysis using a dialysis filter (Micro DispoDialyzer, 1 kDa MWCO Regenerated Cellulose Membrane, Harvard Apparatus). Conjugated GAP27 was incubated with sEVs isolated from LPS-treated slices. The excess of unbounded conjugated GAP27 was removed using a centrifugal concentrator filter (Amicon Ultra-0.5 Centrifugal Filter Unit, 100 kDa MWCO; Merck Millipore).

### 2.11 Statistical analysis

All the datasets underwent the normality analysis (D’Agostino-Pearson omnibus normality test), to assess whether the data were distributed in a Gaussian distribution. On this basis, datasets (all organotypic slice experimental series) were processed with an ordinary one-way ANOVA test to measure statistically significant differences among the treatments (followed by Holm–Sidak comparisons as post-hoc test), otherwise the Kruskal–Wallis test was used (followed by Dunn’s multiple comparison post-hoc test). All the data are expressed as mean ± SEM with n ≥ 3 number of slices from three different culture series unless stated otherwise.

## 3 Results

### 3.1 Isolation of sEVs from control and bzATP-stimulated organotypic spinal cord slices

**Figure 1:**
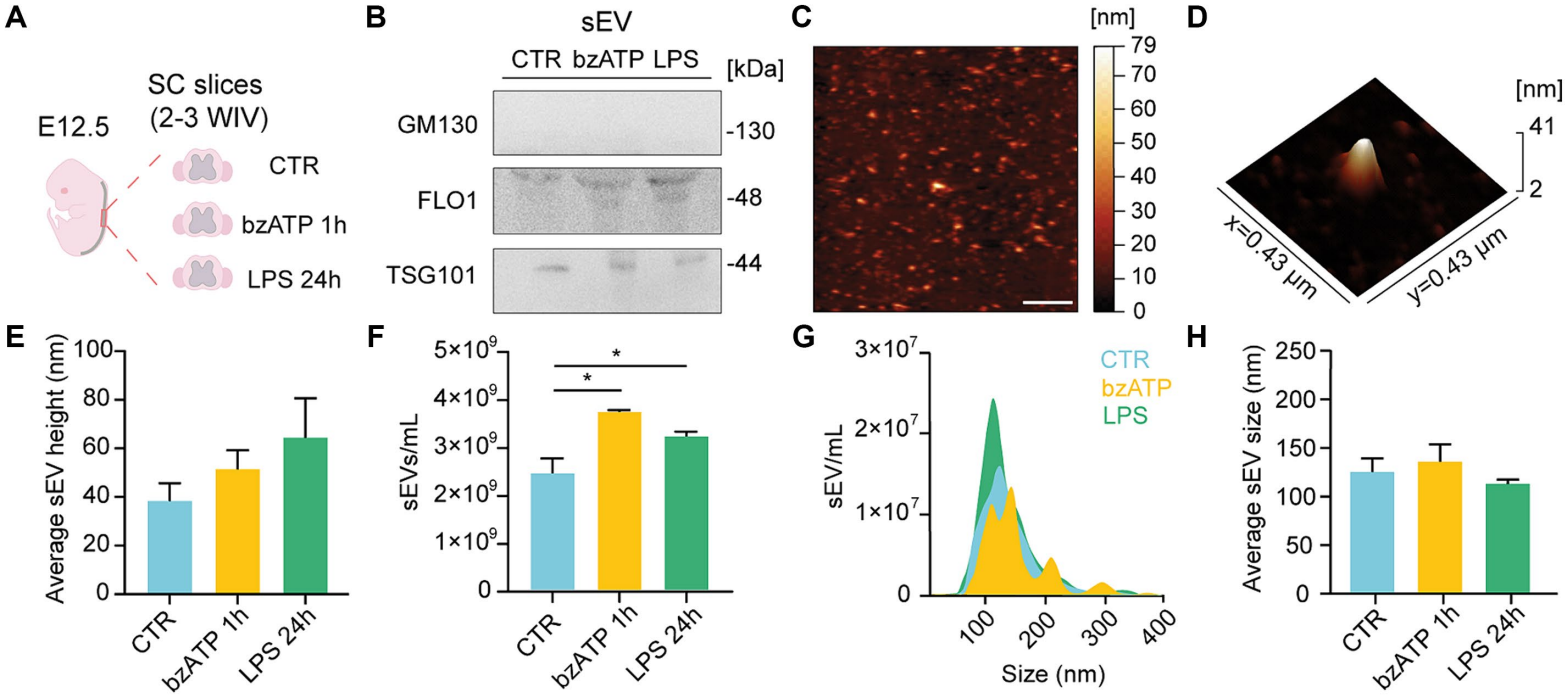
sEV isolation and characterization from organotypic spinal cord slices. **(A)** Experimental procedure overview: slices were isolated from the spinal cord of mice embryos (E12.5) and cultured for 2–3 WIV prior to sEV collection from control (CTR) or bzATP and LPS treatments (1 h and 24 h, respectively). **(B)** Immunoblot analysis of GM130, FLO-1, and TSG101 protein levels obtained from sEVs pellets collected from CTR and bzATP- and LPS-treated slices. **(C)** Representative AFM topographic image of sEV sample obtained after LPS treatment, spotted on mica, dried with N_2_, and imaged in air. Scale bar: 2 μm. **(D)** 3D AFM map of a single sEV spotted on mica. **(E)** Average height of sEVs obtained from CTR and bzATP- and LPS-treated organotypic spinal cord slices. **(F)** NTA quantification of sEVs collected from CTR and bzATP- and LPS-treated organotypic spinal cord slices. *p < 0.05; Kruskal–Wallis test. **(G)** Representative graph of NTA analysis showing the distribution of sEV size for CTR and bzATP- and LPS-treated organotypic spinal cord slices. **(H)** Average sEV size was obtained by NTA and was calculated as mean sEV hydrodynamic radius in CTR and bzATP- and LPS-treated samples. The data from E-H are all tested by the Kruskal–Wallis test.

We used 2–3 WIV organotypic spinal cord cultures (*n* = 12 each treatment) to explore the release of sEVs in three different conditions (shown in Figure 1A): basal condition (control), bzATP to boost the secretion of sEVs (positive secretion control, 100 μM; 1 h) (Doyle and Wang, 2019), and LPS (experimental inflammatory model; 1 μg/mL; 24 h) (Vereker et al., 2000; Li et al., 2003; Panattoni et al., 2021). bzATP and LPS treatments triggered distinct morphological transformations in resident IBA1-positive microglia, when compared with controls (shown in Supplementary Figures S1A,B), in agreement with the development of neuroglia reactivity (Panattoni et al., 2021; Di Mauro et al., 2023).

We collected the supernatant of control and treated slices to isolate sEVs by differential ultracentrifugation (Théry et al., 2006; Musto et al., 2021; see Methods). In Figure 1B, representative WB analysis is shown for the EV biomarker Tumor Susceptibility Gene 101 TSG101 (Gurunathan et al., 2019), flottilin-1 (FLO1; Musto et al., 2021), and the golgi membrane protein GM130 (Sung et al., 2020). The positive presence of TSG101 and FLO1 bands, together with the absence of GM130 ones, in the sEV-resuspended pellets extracted in the control condition and after both bzATP and LPS treatments supports the efficacy of the adopted sEV isolation protocol. The effectiveness of the antibody used to detect GM130 was confirmed by the positive presence of GM130 in the spinal cord lysates that had undergone the same treatments (see Supplementary Figure S1C).

Figures 1C,D show representative AFM images from a sample of sEVs isolated from LPS-treated slices, appearing as vesicle-like objects on the mica surface [(Musto et al., 2021); see Methods]. sEVs mean height, measured by AFM, was similar between control and treated groups (Figure 1E) and within the range of the dimensions expected for sEVs (Parisse et al., 2017; Doyle and Wang, 2019; Pegtel and Gould, 2019).

We further characterized control and bzATP- and LPS-resuspended sEVs by NTA. In the bar plots of Figure 1F, the NTA quantifications of sEVs show a significant increase in sEVs/ml in both bzATP and LPS with respect to control. In Figures 1G,H, the size of sEVs, calculated by NTA measures, is shown as NTA size distribution and mean hydrodynamic radius, respectively, showing the absence of significant differences among groups. The combination of WB, AFM, and NTA approaches positively supported the reliability of the protocol adopted to isolate sEVs in different conditions, showing that both bzATP and LPS treatments were able to trigger the production of sEV.

Additionally, NTA revealed the presence of a minimal amount of residual sEVs in depleted FBS (*n* = 3 samples, from *n* = 3 different FBS stocks) (shown in Supplementary Figure S1E).

### 3.2 LPS-derived sEVs trigger tissue reactivity and astrocyte aberrant calcium dynamic in naïve slices

We incubated (24 h) naïve spinal cord slices (2–3 WIV, *n* = 9 each group) with sEVs isolated from control (sEV_CTR_) or LPS-treated slices (sEV_LPS_), and we used benchmark sister cultures that were maintained in basal conditions (control) or exposed to experimental inflammation (LPS-treated).

**Figure 2:**
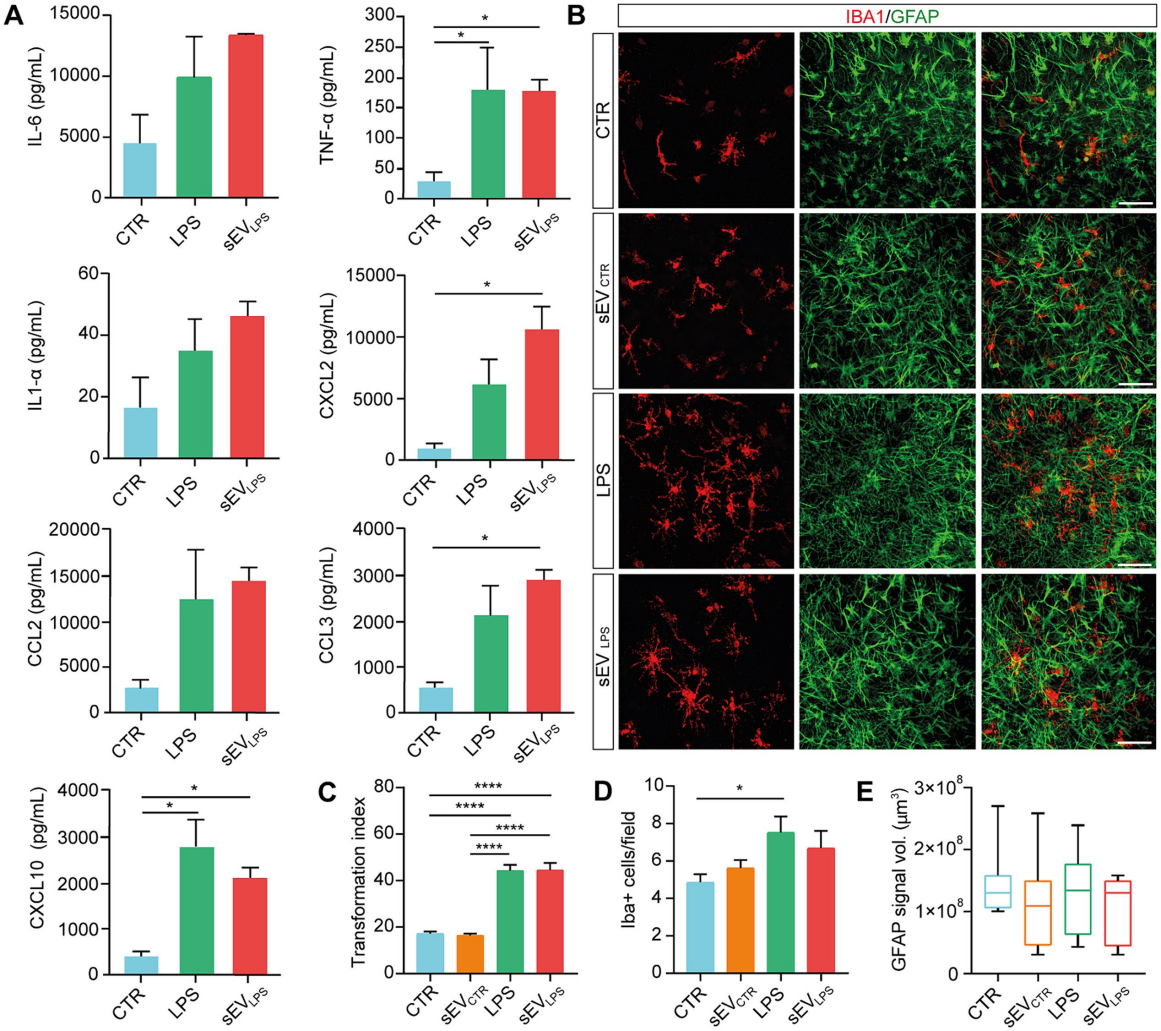
Proinflammatory sEVs induce microglia activation in naïve spinal slices. **(A)** Quantification of cytokines (IL-6; TNF-α; and IL-1α) and chemokines (CXCL2; CCL2; CCL3; and CXCL10) determined by Milliplex assay of supernatants collected from CTR and LPS- and sEV_LPS_- treated organotypic spinal cord slices (*n* = 9 each, from 3 culture series). TNF-α (CTR vs. LPS, *p = 0.0433; CTR vs. sEV_LPS_ *p = 0.0440), CXCL2 (CTR vs. sEV_LPS_ *p = 0.0181), CCL3 (CTR vs. sEV_LPS_ *p = 0.0183), and CXCL10 (CTR vs. LPS *p = 0.0125; CTR vs. sEV_LPS_ *p = 0.0477); Kruskal–Wallis and one-way ANOVA were used. **(B)** Representative confocal micrographs of ventral horn microglia (IBA1, red) and astrocytes (GFAP, green) in organotypic cultures (2–3 WIV) in CTR and slices treated for 24 h with sEVs isolated from control (sEV_CTR_) or LPS or sEVs isolated from LPS-treated slices (sEV_LPS_). Scale bar: 100 μm **(C)** Distribution of IBA1+ cells transformation index (TI) in the different groups analyzed from n = 90, 108, 93, and 54 cells in CTR and sEV_CTR_-, LPS-, and sEV_LPS_-treated slices, respectively. CTR versus LPS and CTR versus sEV_LPS_ ****p < 0.0001; sEV_CTR_ versus LPS and sEV_CTR_ versus sEV_LPS_ ****p < 0.0001; Kruskal–Wallis test was used. **(D)** Quantification of IBA1+ cells/field in all conditions. *n* = 21 fields were analyzed from CTR and sEV_CTR_- and LPS-treated slices, and *n* = 19 fields were analyzed from sEV_LPS_-treated slices. CTR versus LPS *p = 0.033; One-way ANOVA was used. **(E)** Box-plot reporting the volume occupied by GFAP signal in the z-stack and expressed in μm^3^ (*n* = 9 fields). The Kruskal–Wallis test was used.

We quantified the secretion of proinflammatory cytokines and chemokines by Milliplex assay (Panattoni et al., 2021), comparing the supernatant of organotypic slices harvested from control slices and those treated with LPS and sEV_LPS_ (Figure 2A). It is interesting to note that sEV_LPS_ treatment of naïve slices induced an increase, with respect to control, in proinflammatory cytokines and chemokines secretion comparable to LPS treatment. Although this increase did not reach statistical significance in all the considered cases, we detected an explicit positive trend when LPS and sEV_LPS_ were compared with controls, becoming statistically significant for TNF-α and CXCL10, while a statistically significant difference was observed when sEV_LPS_ was compared with control in the cases of CXCL2 and CCL3.

During inflammation, activated glial cells modify their phenotype by showing changes in cell number, morphology, and function (Giacco et al., 2019; Refolo and Stefanova, 2019; Panattoni et al., 2021). In all four culture groups, we visualized microglia and astrocytes by co-immunolabeling cultured spinal slices with the specific cytoskeletal markers IBA1 and GFAP (Figure 2B; Giacco et al., 2019). LPS incubation induced changes in microglia ramification, which was usually associated with one of the different stages of microglia activation (Kreutzberg, 1996; Perry et al., 2007). LPS stimulated an activated ramified morphology with long and thick processes (Mathieu et al., 2010; Giacco et al., 2019), which is correlated with a large TI index, as shown in Figure 2C, together with an increase in IBA1+ cell/field with no modifications in GFAP signal (summarized in Figures 2D,E); similar changes were promoted by sEV_LPS,_ while sEV_CTR_ recapitulated control features.

To rule out the influence of soluble mediators such as residual LPS, naïve slices (n = 8 per condition) were incubated for 24 h with: *i.* the residual medium *per se* depleted of sEVs upon centrifugation (the supernatant usually discarded after isolation of sEV_LPS_, “sEV_LPS_ residual medium”); *ii.* sEV_LPS_ (resuspended in fresh medium, “sEV_LPS_ resuspended in fresh medium”), and *iii.* The fresh resuspension medium, once again deprived of sEV_LPS_ (“sEV_LPS_ residual fresh medium”), as shown in Supplementary Figure S1F. All treatments were compared with the control. IBA1+ cell TI (taken as a proxy of inflammation) was affected by the residual medium and sEV_LPS_ but not by the residual fresh medium (Supplementary Figures S1G,H), excluding that the presence of soluble factors contaminating sEV suspension might be responsible for sEVs_LPS_ biological activity.

**Figure 3:**
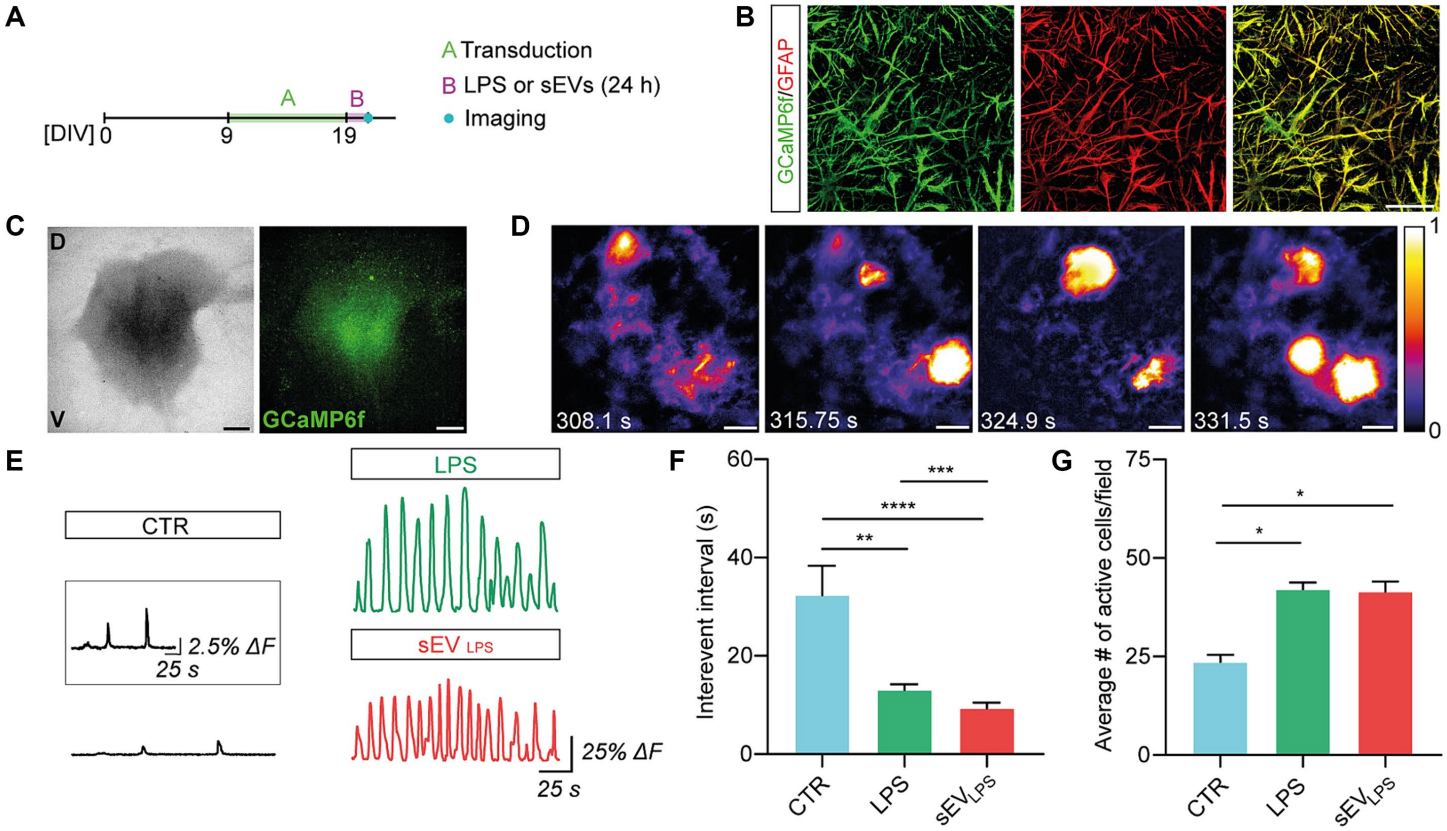
Proinflammatory sEVs induce dysfunctional calcium dynamics in astrocytes. **(A)** Sketched experimental time line. Organotypic slices were cultured for 9 days and then infected with AAV5.gfaABC1D-cyto-GCaMP6f able to transduce GFAP+ astrocytes. After 10 days and upon 24 h CTR, LPS, and sEV_LPS_ treatments, live calcium imaging was performed. **(B)** Representative confocal micrographs showing the colocalization of GCaMP6f (green) and GFAP (red) confirming the selective expression of GFP-based calcium sensor in GFAP+ astrocytes. Scale bar: 50 μm. **(C)** Representative low-magnification brightfield (left) and confocal (right) images of the organotypic spinal slice infected with AAV5.gfaABC1D-cyto-GCaMP6f (green). V: ventral horn, D: dorsal horn. Scale bar: 400 μm. **(D)** Frames from the live calcium imaging recording of a control slice at different time points showing the characteristic astrocytic calcium events. Intensity calibration was performed, and the scale was reported. Scale bar 100 μm. **(E)** Representative fluorescence tracing showing ΔF over time in CTR and LPS- and sEV_LPS_-treated slices. Scale bar: 25 s, ΔF = 25%. The inset shows a magnification of the CTR Ca^2+^ signal. Scale bar: 25 s, ΔF = 2.5%. **(F)** Distribution of interevent intervals (IEI) in control and LPS- and sEV_LPS_-treated slices (*n* = 6 each condition). CTR versus LPS, **p = 0.0012; CTR versus sEV_LPS_ ****p < 0.0001; LPS versus sEV_LPS_ ***p = 0.0002; Kruskal–Wallis test was used. **(G)** Distribution of the average number of active astrocyte/field in CTR and LPS- and sEVs_LPS_-treated slices (*n* ≥ 4 fields per condition). CTR versus LPS, *p = 0.0301 and CTR versus sEV_LPS_, *p = 0.0451; the Kruskal–Wallis test was used.

CNS astrocytes generate changes in cytoplasmic calcium concentrations to propagate signals, integrate network functional states, and promote the release of gliotransmitters (Bindocci et al., 2017; Semyanov et al., 2020). Inflammatory molecules may trigger aberrant calcium signals in reactive astrocytes (Shigetomi et al., 2019), which, in turn, contribute to neuroinflammatory states. Physiological and non-physiological astrocytic Ca^2+^ elevations are replicated in spinal organotypic slices (Panattoni et al., 2021; Di Mauro et al., 2023). We monitored live calcium responses from astrocytes in control and LPS and sEV_LPS_-cultured spinal explants (*n* = 6 for each group; 3 WIV) by GCaMP6f GECIs based on green fluorescent protein (GFP), following the protocol shown in Figure 3A. Calcium sensor expression was restricted to astrocytes (Haustein et al., 2014), as confirmed by co-immunolabeling for GFAP and GCaMP6f (Figure 3B). Figure 3C shows low-magnification images of organotypic culture (bright field, left) and the GCaMP6f signal (right) from the same slice. As shown in Figure 3D, high-magnification time-lapse from the sampled ventral area (680.42 × 680.42 μm^2^) is visualized, and in Figure 3E, sample fluorescence tracing depicts intracellular calcium dynamics in astrocytes comparing control, LPS, and sEV_LPS_ cultures. In resting conditions (control), astrocytes displayed irregular calcium oscillations occurring at a low pace, with a mean inter-event interval (IEI) significantly higher than that measured in LPS- and sEV_LPS_-treated cultures, where lower time intervals indicate an increased occurrence of calcium events (Figure 3F). Interestingly, sEV_LPS_ treatment induces an additional significant reduction in IEI compared with the LPS condition (Figure 3F; Panattoni et al., 2021; Di Mauro et al., 2023). Accordingly, a significantly higher number of active astrocytes were measured in both reactive conditions compared with control (Figure 3G).

These results suggested a specific and crucial role of sEV_LPS_ in altering microglia and morphology and physiology of astrocytes, with the emergence of aberrant calcium signals in GFAP+ cells.

### 3.3 Proinflammatory sEVs induce altered calcium dynamics by increasing hemichannel permeability

**Figure 4:**
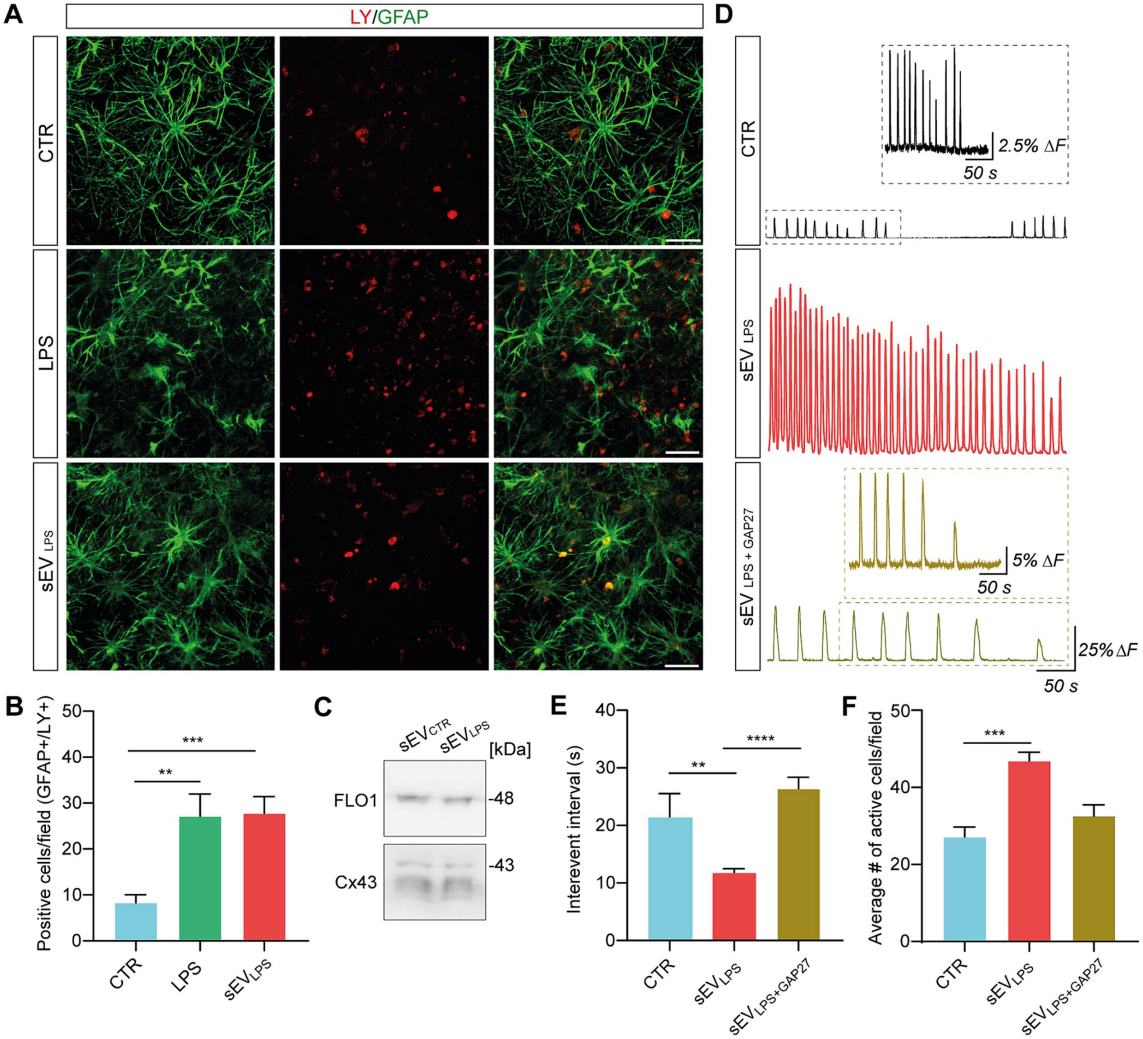
Proinflammatory sEVs altered calcium dynamics through a Cx43-dependent mechanism. **(A)** Representative confocal micrograph showing LY (in red) uptake in GFAP+ cells (in green) in CTR and LPS- and sEV_LPS_-treated slices. Scale bar: 50 μm. **(B)** Quantification of double positive GFAP/LY cells in CTR (*n* = 22 fields) and LPS- (*n* = 28 fields) and sEV_LPS_ (*n* = 28 fields)-treated slices (*n* = 8 each condition). CTR versus LPS **p = 0.0093; CTR versus sEVs_LPS_ ***p = 0.0005; the Kruskal–Wallis test was used. **(C)** Western blot of sEV_CTR_ and sEVs_LPS_ pellets showing the presence of vesicles-associated Cx43 (all the isoforms: 43, 40, 37, and 36 kDa). FLO-1 was used as a sEV marker. **(D)** Fluorescent tracings in CTR (black), sEV_LPS_ (red), and sEV_LPS + GAP27_ (ochre) astrocytes. Scale bar: 50 s, ΔF =25%. The insets show a magnification of the CTR and sEV_LPS + GAP27_ Ca^2+^ signal, respectively. Scale bar: 25 s; ΔF = 2.5% for CTR and 5% for sEV_LPS + GAP27_, respectively. **(E)** Distribution of interevent intervals (IEI) in CTR and sEV_LPS_- and sEV_LPS + GAP27_-treated slices (*n* = 6 each condition). CTR versus sEV_LPS_ **p = 0.0037; sEV_LPS_ versus sEV_LPS_ + _GAP27_ ****p < 0.0001; the Kruskal–Wallis test was used. **(F)** Quantification of the average number of active astrocytes per field in CTR and sEV_LPS_- and sEV_LPS + GAP27_-treated slices (*n* = 7 fields each condition); CTR versus sEVs_LPS_ ***p = 0.0006, the Kruskal–Wallis test was used.

In astrocytes, the pathological increase in HC membrane permeability contributes to inflammation spreading and aberrant calcium dynamics (Orellana et al., 2016; Panattoni et al., 2021; Di Mauro et al., 2023). We investigated HC permeability by quantifying Lucifer Yellow (LY; 1 mM) dye uptake (Kanaporis et al., 2011; Di Mauro et al., 2023) in GFAP^+^ astrocytes in control, LPS, and sEV_LPS_ (n = 8 each group). In Figure 4A, representative micrographs show LY- and GFAP-positive cells, and double-positive cells are quantified in the bar plot, as shown in Figure 4B. LPS and sEV_LPS_ conditions significantly increased the number of double LY-GFAP-positive cells. In an additional set of experiments, we addressed the major pathway of LY uptake in astrocytes by GAP27, a mimetic peptide known to block HC opening triggered by exogenous stimuli (Wang et al., 2012). By selectively blocking Cx43 HCs, which were abundantly expressed in astrocytes with GAP27 peptide after LPS treatment, we observed that the enhanced LY uptake brought about by LPS returned to levels comparable to the controls (see Supplementary Figures S2A,B). This result suggests that Cx43 HC plays a key role in LY uptake in astrocytes and, consequently, in the regulation of calcium signals.

sEVs are known to carry Cx43 HCs on their vesicle membrane (Soares et al., 2015), and translocating Cx43 HCs with enhanced permeability might be an upstream key trigger to expand inflammation and activate astrocytes. Once the presence of Cx43 in sEV_CTR_ and sEV_LPS_ lysates was confirmed by WB (Figure 4C; Supplementary Figure S1I), the involvement of Cx43 HC in spreading inflammation was investigated. Thus, the calcium dynamics of astrocytes was assessed after 24 h of incubation with sEV_LPS_ or sEV_LPS + GAP27_ as compared with the control condition (*n* = 6 slices per condition). sEV_LPS + GAP27_ complex was obtained by incubating sEV_LPS_ with GAP27 (500 μM; 30 min; 4°C), washing out the unbound peptide and resuspending the complex in fresh medium prior to the treatment of the slices. Unblocked sEV_LPS_ were subjected to the same procedures for consistency. Figure 4D shows representative fluorescence tracing, where astrocytic calcium transients were significantly increased upon sEV_LPS_ treatment when compared with control; conversely, sEV_LPS + GAP27_ -treated samples exhibit calcium dynamics in control conditions. In accordance with these measures, a significant reduction in the astrocytic IEI and an increase in the number of active cells/field were observed only after sEV_LPS_ treatment (Figures 4E,F).

**Figure 5:**
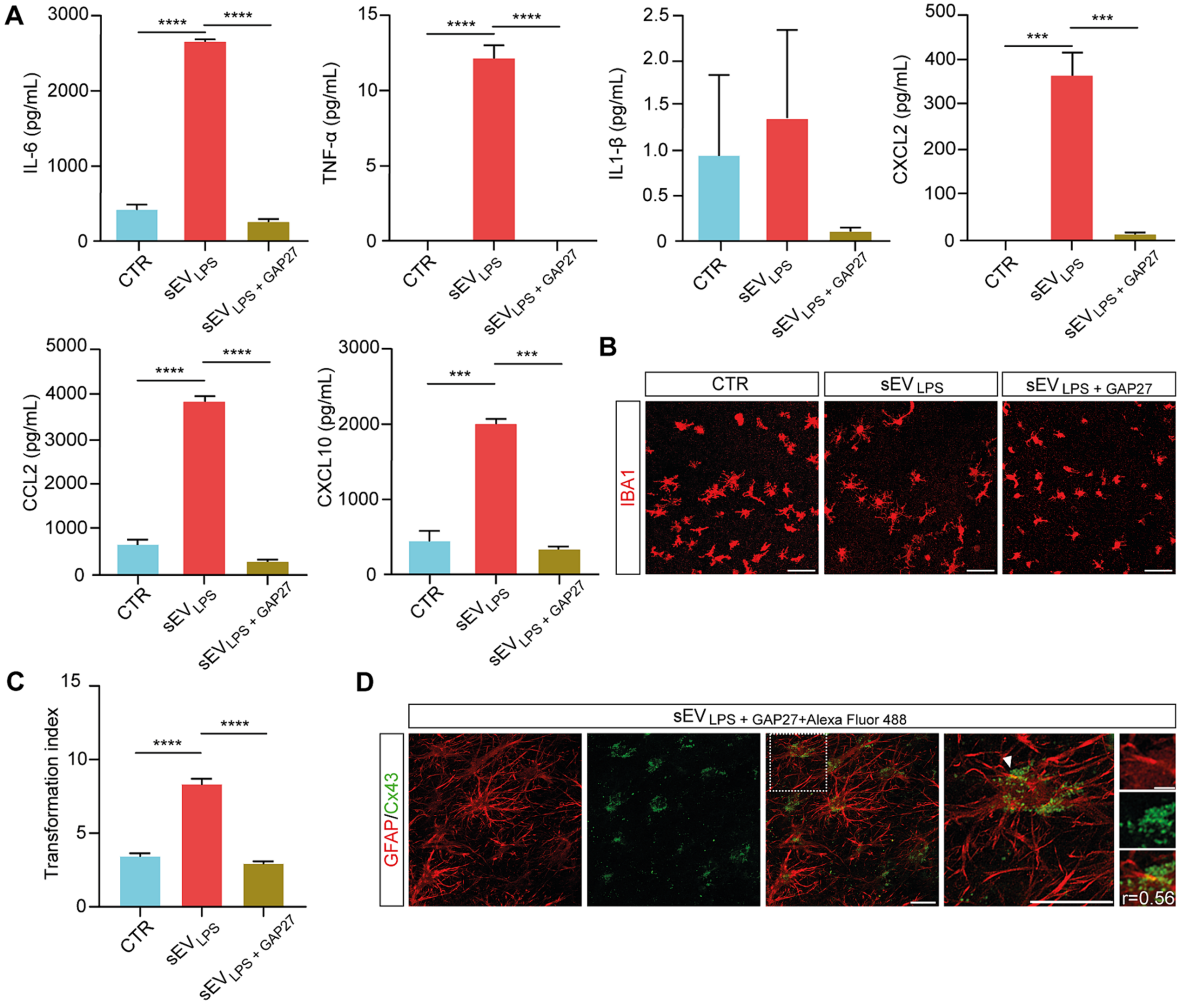
Selective inhibition of HCs in sEVs_LPS_ suppresses inflammatory spreading to naïve treated slices **(A)** Quantification of secreted cytokines (IL-6; TNF-α; and IL-1β) and chemokines (CXCL2; CCL2; and CXCL10) determined by Milliplex assay of supernatants collected from CTR and sEV_LPS_- and sEV_LPS + GAP27_-treated organotypic slices. TNF-α (CTR versus sEV_LPS_ and sEVs_LPS_ versus sEV_LPS+ GAP27_ ****p < 0.0001); IL-6 (CTR versus sEV_LPS_ and sEV_LPS_ versus sEV_LPS+ GAP27_ ****p < 0.0001); CXCL2 (CTR versus sEV_LPS_ ***p < 0.0002, sEV_LPS_ versus sEV_LPS+ GAP27_ ***p < 0.0002); CCL2 (CTR versus sEV_LPS_ and sEV_LPS_ versus sEV_LPS+ GAP27_ ****p < 0.0001); CXCL10 (CTR versus sEV_LPS_ *** *p* < 0.0005, sEV_LPS_ versus sEV_LPS+ GAP27_ ***p < 0.0004.); Kruskal–Wallis and one-way ANOVA tests were used. **(B)** Representative confocal micrograph of microglia marked with anti-IBA1 (red) before and after incubation with sEV_LPS_ and sEV_LPS + GAP27_. Scale bar: 100 μm. **(C)** Distribution of IBA1+ cells TI in the different groups analyzed from n = 86, 220, 174 cells of CTR and sEV_LPS_- and sEV_LPS + GAP27_-treated slices, respectively. CTR versus sEV_LPS_ and sEV_LPS_ versus sEV_LPS+ GAP27_ ****p < 0.0001; the Kruskal–Wallis test was used. **(D)** Representative confocal micrograph showing the colocalization between GFAP+ astrocytes (red, left) and sEV_LPS_ carrying Cx43 tagged with GAP27 + Alexa Fluor 488 NHS Ester (green, middle; merged in middle right panel). Scale bar: 30 μm. Right, magnification of merged panel (dotted white square), the white arrows point at Cx43 (green dots) co-localized with GFAP+ cell soma. Scale bar: 30 μm. Insets of the selected region show at high-magnification GFAP and Cx43 signals, where Pearson’s correlation coefficient (r) was calculated. Scale bar: 5 μm.

By Milliplex assay of the supernatants harvested from control, sEV_LPS_, and sEV_LPS + GAP27_, we evaluated the inflammatory status of the treated slices. Interestingly, the concentration of cytokines and chemokines in the supernatant of sEV_LPS + GAP27_ was comparable to control and significantly lower when compared with that of sEV_LPS_-treated samples (Figure 5A). Milliplex results were strengthened by microglia TI values in sEV_LPS + GAP27_-treated slices, which were comparable to control and significantly lower in sEV_LPS_-treated samples (shown in Figure 5B and quantified in Figure 5C).

To selectively track the localization of sEVs in the slices, sEV_LPS + GAP27 + Alexa Fluor 488_ was used. In brief, GAP27 was pre-incubated with Alexa Fluor 488 NHS Ester (see Methods), a fluorescent dye able to bind to the primary amines (R-NH_2_) of the peptide. After removing the unbound dye using a dialysis filter (1 kDa MWCO; see Methods), the complex GAP27 + Alexa Fluor 488 NHS ester was further incubated with sEV_LPS_ (1 h, 4°C). After removing the unbound tagged GAP27 by centrifugal filter unit (100 kDa MWCO; see Methods), sEVs_LPS + GAP27 + Alexa Fluor 488_ were isolated, carrying Cx43 HCs tagged with GAP27-Alexa Fluor 488 NHS Ester complex. sEVs_LPS + GAP27 + Alexa Fluor 488_ were used to treat spinal organotypic slices [*n* = 3, 1 h, as recommended in the study by Jurgielewicz et al. (2020) and Saffitz et al. (2000)], and the immunolabeling with anti-GFAP antibody confirmed the co-localization between Alexa Fluor 488 NHS Ester spots and GFAP+ astrocytes (Figure 5D). Although preliminary, this result supports a direct interaction between astrocytes and HCs carried by sEV_LPS_.

## 4 Discussion

In this study, we successfully isolated sEVs released by spinal cord organ cultures under resting or inflammatory reactive states. We also report the ability of sEVs, when delivered by activated cells, to spread neuroinflammation in naïve tissues by triggering reactive morphological and functional features in exposed microglia and astrocytes.

The procedure to extract sEVs from experimental or clinical biological samples is still a matter of debate, with purification of sEVs representing one of the major challenges in this area of investigation (Patel et al., 2019; Sidhom et al., 2020). The current lack of standard procedures to isolate sEVs has prompted the proposal of new isolating technologies, particularly relevant when exploiting sEVs as disease biomarkers (Chiriacò et al., 2018; Moon et al., 2019; Perissinotto et al., 2020). To isolate sEVs released by organotypic spinal cord slices, we adopted a largely used protocol based on ultracentrifugation (Jeppesen et al., 2019), which *per se* cannot rule out the potential presence of contaminants or heterogeneous vesicle subpopulations (Marostica et al., 2021). However, our AFM, NTA, and WB results supported the presence of a relatively homogenous sEV population in terms of vesicle size, although we cannot exclude small vesicles released by membrane budding (Chen et al., 2022). Being aware of the potential of sEV of mixed endosomal/not-endosomal origin, we are confident with their belonging to the small vesicle population, which is extracted in the absence of significant contaminations from the spinal-cultured tissue, as supported by the blot and the control experiments adopting the re-suspended medium. In addition, the danger paradigm used leads to mild neuroinflammation, as confirmed in our histological and functional experiments, with negligible cell death (Panattoni et al., 2021). We also reported a negligible amount of vesicle in FBS-depleted serum, which, together with the absence of detectable changes in slice resident glia when treated by sEVs_CTR_, further indicating the genuine spinal tissue origin of the isolated sEVs.

Organotypic slice cultures provide optimal experimental settings to reproduce inflammatory microenvironments, which have been previously described (Giacco et al., 2019; Panattoni et al., 2021; Di Mauro et al., 2023). LPS treatments activate an immune condition, where LPS binds to the TLRs expressed on the microglia surface resulting in the production and release of cytokines, chemokines, and other inflammatory factors (Panattoni et al., 2021). Similarly, in the current results, the changes in microglia ramification status in the absence of clear GFAP hypertrophy (Giacco et al., 2019), but accompanied by the emergence of increased active astrocytes and characterized by paroxysmal, aberrant calcium signaling (Panattoni et al., 2021), are good indicators of the build-up of local inflammation, which are triggered by LPS or re-vamped by sEVs_LPS_, further supported by the detection of cytokines and chemokines. We monitored astrocyte live calcium activity without the need for pharmacological removal of neuronal activity (Panattoni et al., 2021), since GCaMP6f allows precise identification of active phenotypes while maintaining the background synaptic activity. Indeed, since inflammatory conditions enhance spinal synaptic activity (Medelin et al., 2008; Di Mauro et al., 2023), and glial cell signaling also reflects neuronal electrical activity, our current experimental conditions may explain the higher amount of spontaneously active cells detected in each field, in resting or immune activated status, when compared with previous studies (Panattoni et al., 2021).

sEVs released and primed by inflammatory activated cells coordinate homeostatic reactivity and induce specific danger-associated changes in resident glial cells. In doing so, sEVs may interact with recipient cells in three major modalities (Zhao et al., 2021), and they may be taken up by endocytic pathways (non-specific or receptor-mediated); sEVs may directly fuse with the plasma membrane or sEVs may remain attached to the plasma membrane in all three cases triggering downstream signaling pathways (Gurung et al., 2021).

We confirmed the presence of Cx43 in sEVs, supporting that they are being mostly released by astrocytes, both at rest and in LPS neuroinflammatory *mileu*. In our experiments, the observed increase in HC permeability upon delivery of sEVs_LPS_ was prevented by the pre-treatment of sEVs_LPS_ with an HC blocker. In 2015, Soares et al. demonstrated that Cx43 HCs were present at the level of sEV membrane and were essential for cargo transfer to recipient cells (Soares et al., 2015). In our experimental model, blocking open HC by GAP27 in sEVs_LPS_ fully prevented the induction of inflammation in naïve slices. A potential mechanism might involve the formation of gap junctions between astrocytic and sEVssomal Cx43 HCs, allowing pro-inflammatory cargo transfer to the recipient cells (Soares et al., 2015), with astrocyte reactivity leading to HC opening (Panattoni et al., 2021). An alternative mechanism might involve vesicle-directed fusion and the active transfer of open and functional Cx43 HCs from sEVs to the astrocytic plasma membrane. In this scenario, enhancing the permeability of the astrocyte HCs would promote the release of pro-inflammatory factors contributing to CNS damage progression (Makarenkova and Shestopalov, 2014; Bernaus et al., 2020; Peng et al., 2022). Further experiments are needed to identify sEV target cells and mechanisms of action, it is interesting to note that our preliminary results tracking sEV HCs indicated the transfer of sEVs_LPS_ from HC to astrocytes.

### 4.1 Scope statement

Neuroinflammation is a fundamental component of neurodegenerative diseases, and it involves the activation of resident microglia and astrocytes. Resident cells, by regulating CNS inflammatory responses, may exacerbate CNS reactivity and neurodegeneration progress. In the CNS, extracellular vesicles (EVs), namely bilayer membrane vesicles released by neurons and glial cells, are key mediators of intercellular signaling and CNS homeostasis. EVs may also mediate neuroinflammatory responses and promote tissue damage.

We addressed the role of EVs released by activated CNS cells in propagating reactivity to healthy tissue, supporting the negative spiral that escalates neuroinflammation.

We used organ spinal cord cultures to mimic CNS resting and reactive states and harvest EVs released by spinal resident cells. Naïve slices were then exposed to the resting or reactive EVs to elucidate the impact of vesicles on spreading local inflammation in the spinal cord. We propose a distinct role of EVs in delivering proinflammatory signaling to resting astrocytes, and we further hypothesize a specific interaction between EV hemichannels and astrocyte membrane, crucially regulating astrocyte calcium dynamics and spinal cord reactivity.

Our study provides new insights into the role of EVs in regulating CNS local tissue reactivity, ultimately affecting astrocyte calcium dynamics and microglia release of cytokines and chemokines.

## Data availability statement

The raw data supporting the conclusions of this article will be made available by the authors, without undue reservation.

## Ethics statement

Animal use was approved by the Italian Ministry of Health (no. 22DABNQYA and 22DABN1WO), in agreement with the EU Recommendation 2007/526/CE. The study was conducted in accordance with the local legislation and institutional requirements.

## Author contributions

CM: Formal analysis, Investigation, Methodology, Writing – original draft. PP: Formal analysis, Investigation, Methodology, Writing – review & editing. RA: Formal analysis, Investigation, Methodology, Writing – original draft. MP: Data curation, Formal analysis, Validation, Writing – review & editing. AP: Data curation, Investigation, Validation, Writing – review & editing. LC: Conceptualization, Resources, Writing – review & editing. CB: Conceptualization, Funding acquisition, Writing – review & editing. LB: Conceptualization, Funding acquisition, Writing – review & editing.
