# Supplementary material for: Extracellular vesicles released by LPS-stimulated spinal organotypic slices spread neuroinflammation into naïve slices through connexin43 hemichannel opening and astrocyte aberrant calcium dynamics
Source: Front Cell Neurosci. 2024 Jul 10;18:1433309. doi: 10.3389/fncel.2024.1433309 (PMC11266295; doi:10.3389/fncel.2024.1433309)
Supplement: Supplementary file 1 [file Data_Sheet_1.docx]

Supplementary Material

# Supplementary Figures and Tables

## Supplementary Figure 1

­­­


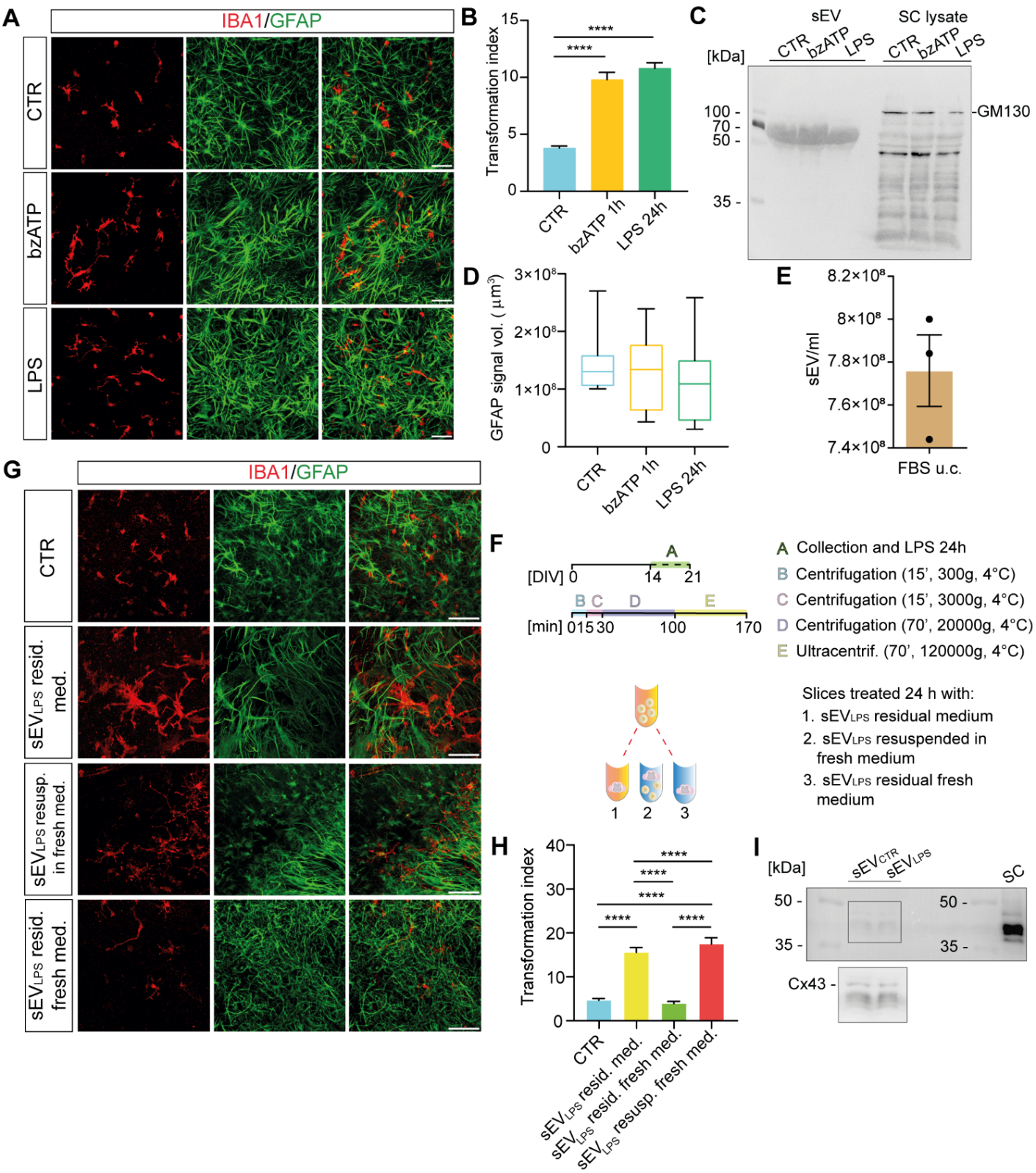


**Supplementary Figure 1.** **(A)** Representative confocal micrograph of ventral horn showing IBA+ cells (in red) and GFAP+ cells (in green) in control organotypic slices (2-3 WIV), and after bzATP and LPS treatments. Scale bar: 50 µm. **(B)** Quantification of IBA1+ cells TI analyzed from n = 163, 130, 158 cells from control, bzATP -and LPS-treated slices, respectively. CTR vs bzATP and CTR vs LPS, ****p < 0.0001; Kruskal-Wallis test was used. **(C)** Whole Western Blot membrane reporting the expression of GM130 protein obtained from sEVs pellets collected from CTR, bzATP- and LPS-treated slices, and from CTR, bzATP- and LPS-treated spinal cord cultures. **(D)** Box-plot reporting the volume occupied by GFAP signal in the z-stack expressed in µm3 (n = 9 fields); Kruskal-Wallis test was used. **(E)** Quantification of the sEV concentration (sEV/mL) in n = 3 samples of sEV-depleted FBS serum. **(F)** Protocol overview: slices were treated with LPS for 24 h to promote sEVs release. sEVs-residual medium, sEVs-resuspended in fresh medium and sEVs-residual fresh medium were used to treat naïve slices for 24 h. **(G)** Representative confocal micrograph of IBA+ cells (in red) and GFAP+ cells (in green) in the ventral horn of spinal slices (2-3 WIV) in control or treated with: sEV-residual medium, sEV_LPS_ resuspended in fresh medium and sEV-residual fresh medium. Scale bar: 50 µm. **(H)** Quantification of IBA+ cells TI analyzed from n = 39, 32, 47 and 23 cells from CTR and from slices treated with: sEV-residual medium, sEV resuspended in fresh medium and with sEV-residual fresh medium, respectively. CTR vs sEV_LPS_ resid. medium, CTR vs sEV_LPS_ resusp. in fresh medium, sEV_LPS_ resid. medium vs sEV_LPS_ resid. fresh medium, sEV_LPS_-resid. medium vs sEV_LPS_ resusp. in fresh medium, ****p < 0.0001; Kruskal-Wallis test was used. **(I)** Immunoblot analysis of Cx43 protein levels obtained from sEVs pellets collected from CTR and LPS-treated slices. The inset is showing a zoom of the connexin bands of interest. Spinal cord lysate was used as a positive control of the expression of Cx43 and its isoforms.

##
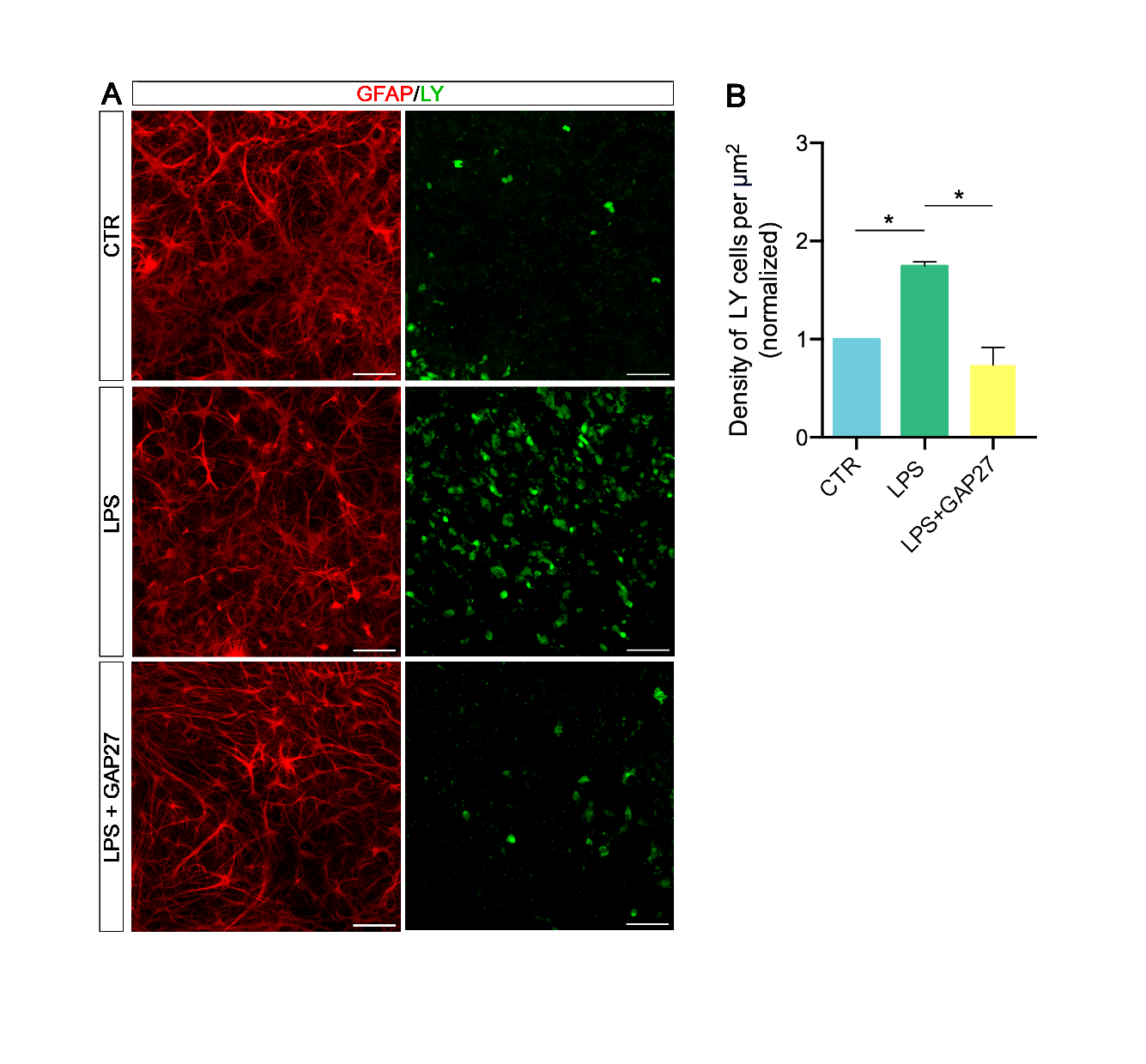
Supplementary Figure 2

**Supplementary Figure 2.** **(A)** Representative confocal micrograph showing LY (green) uptake and GFAP+ cells (red) in CTR, LPS, and LPS+GAP27-treated slices. Scale bar: 50 µm. **(B)** Quantification of LY uptake cell density normalized to CTR values, *p < 0.05; Two-tailed statistical unpaired t test was used.
